# Supplementary material for: Higher resolution pooled genome-wide CRISPR knockout screening in Drosophila cells using integration and anti-CRISPR (IntAC)
Source: Nat Commun. 2025 Jul 15;16:6498. doi: 10.1038/s41467-025-61692-3 (PMC12259957; doi:10.1038/s41467-025-61692-3)
Supplement: Supplementary file 1 — Supplementary Information [file 41467_2025_61692_MOESM1_ESM.pdf]

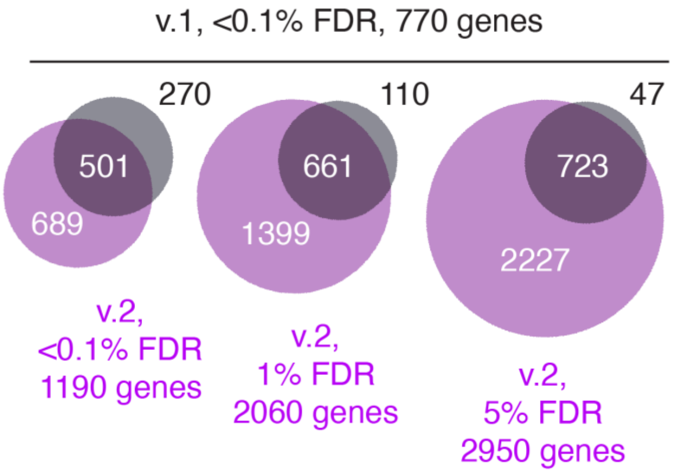

Supplementary Figure 1: Comparison of v.1 and v.2 screens. The 770 genes with the strongest likelihood of representing bona fide fitness genes in v.1 screens are mostly a subset of those detected in v.2. The overlap becomes progressively clearer when the false-discovery rate (FDR) of v.2 fitness genes is relaxed.

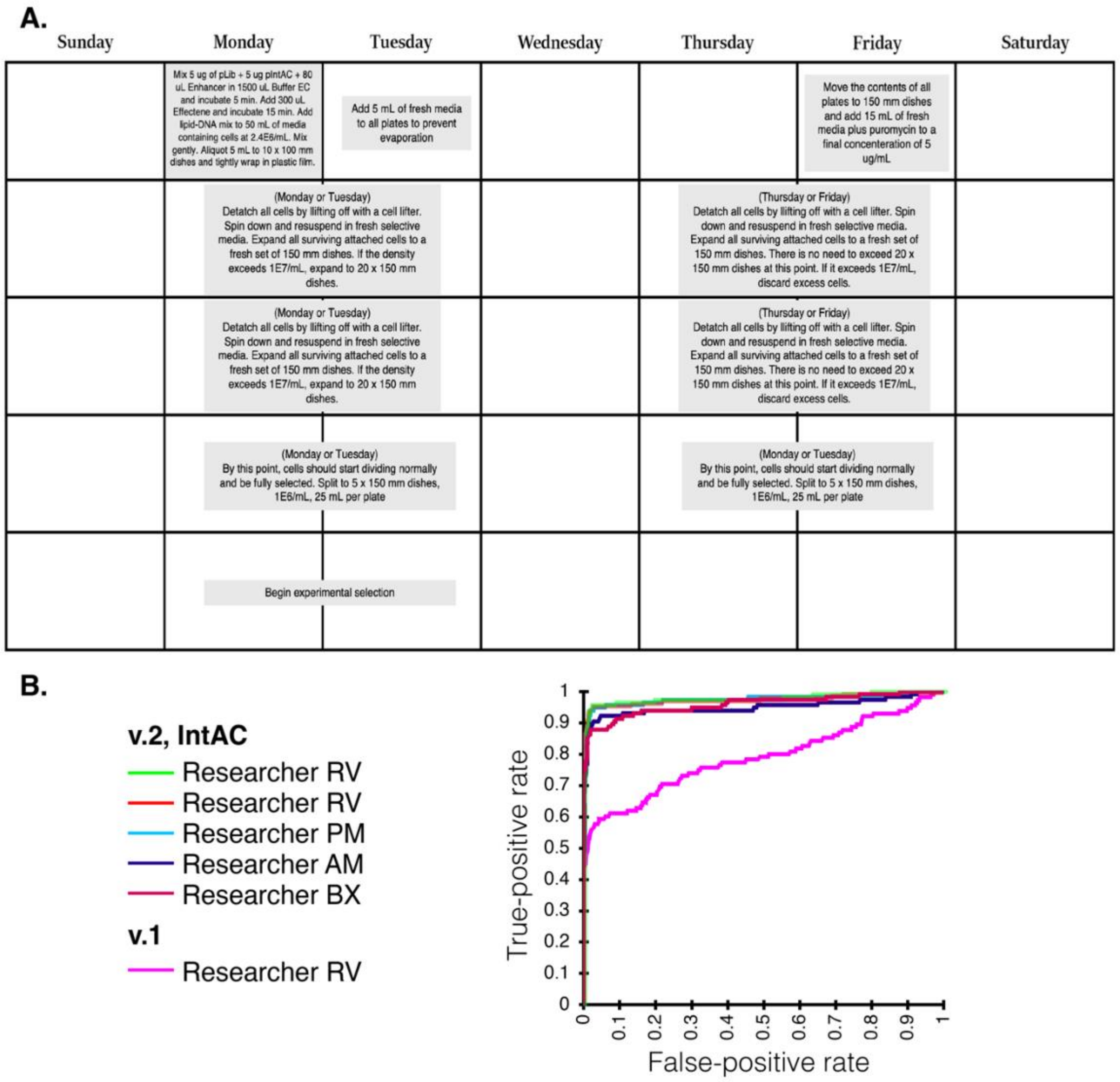

Supplementary Figure 2: Validation of IntAC CRISPR screen across independent trials and researchers. (A) Hands-on steps for generating a pooled library using IntAC. (B) Performance of the IntAC platform in independent trials by different researchers. Data from multiple independent screens conducted using the IntAC platform shows high reproducibility in terms of sgRNA dropout patterns and gene Z-scores. A precision-recall analysis comparing the detection of essential genes (true-positives, KEGG-assigned ribosome and proteasome genes) versus false-positives (non-expressed genes, genes with mRNA FPKM < 1). These independent trials confirmed the high precision and robustness of the v.2, IntAC, system relative to the previous v.1 CRISPR screening system in the hands of different researchers

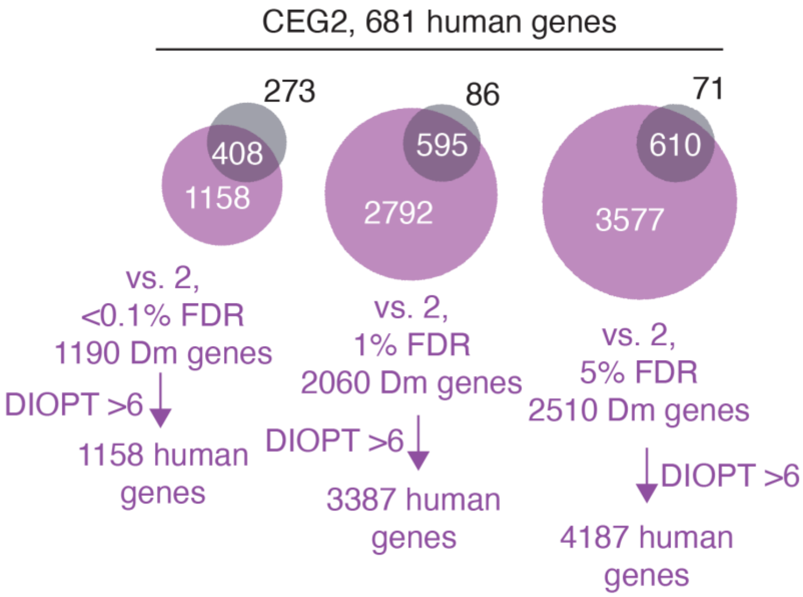

Supplementary Figure 3: Overlap between human cell essential geneset and *Drosophila* cell essential geneset. The 681 genes in the core essential geneset v.2 [67]. The overlap becomes progressively clearer when the false-discovery rate (FDR) of v.2 fitness genes is relaxed.

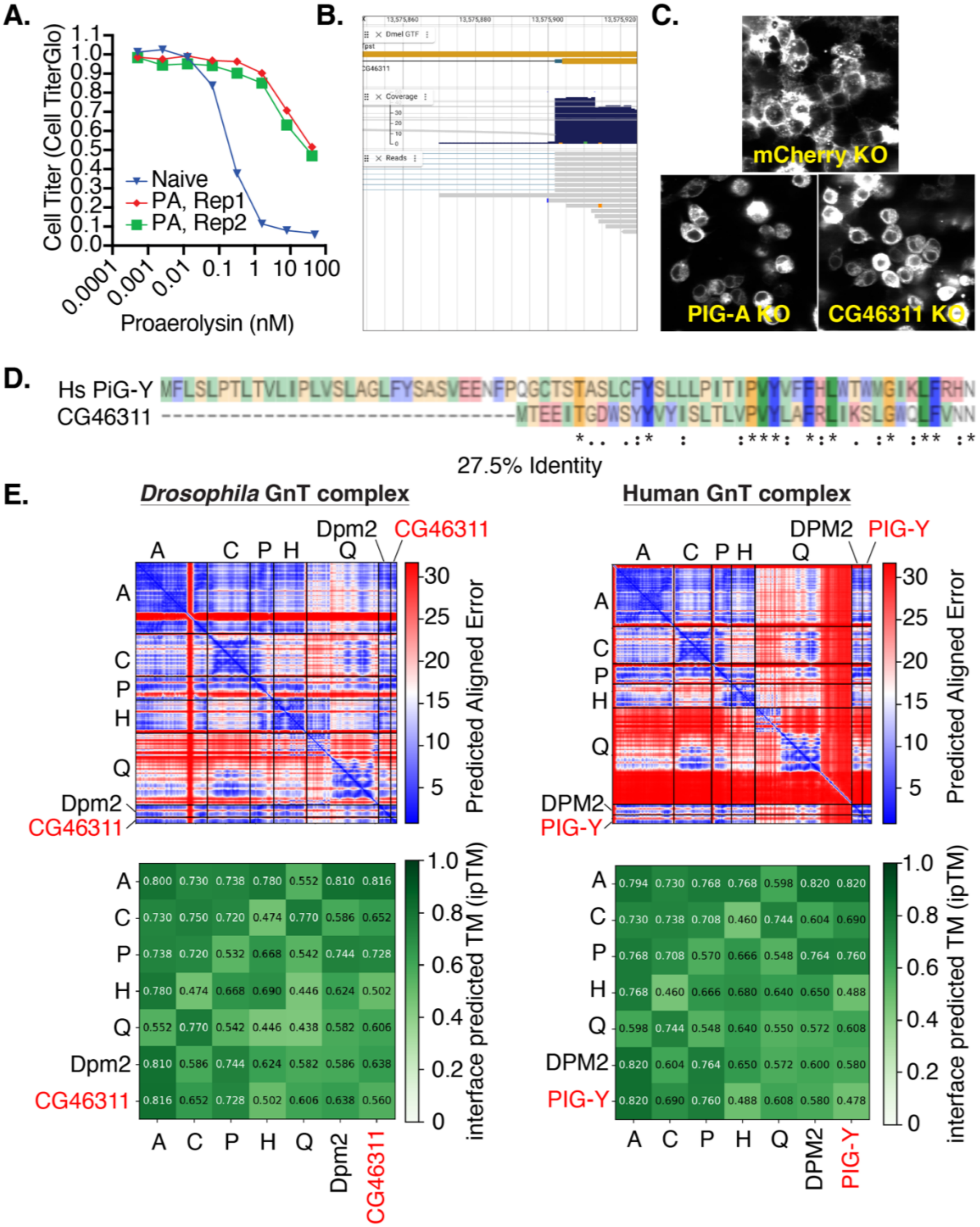

Supplementary Figure 4: A proaerolysin (PA)-resistance screen identifies a small ORF (CG46311) as a novel regulator of the GPI synthesis pathway, likely the "missing" ortholog of human PIG-Y. (A) Cell Titer Glo viability assay with varying dosage of PA demonstrating that following three weeks of treatment of an IntAC library

with 2 nM PA, surviving cells gained 10-100-fold resistance to PA killing. Experiment was not replicated (N=1). (B) S2R+ RNAseq data illustrates expression of CG46311. (C) Detection of GFP-tagged GPI reporter (GFP-HsCD58^{GPI}) in wild-type cells (expressing a control sgRNA targeting mCherry), PIG-A knockout cells, or CG46311 KO cells demonstrating that PIG-A or CG46311 knockout leads to internal GFP-HsCD58^{GPI}. Representative image of three experimental replicates. (D) Sequence alignment between CG46311 and human PIG-Y shows low but detectable protein identity. (E) Alignment metrics (predicted aligned error, PAE, and interface predicted template modeling, ipTM) between the polypeptides within the Alphafold-Multimer (Alphafold3) predicted GnT complexes from *Drosophila* or humans.
