# Supplementary material for: Higher resolution pooled genome-wide CRISPR knockout screening in Drosophila cells using integration and anti-CRISPR (IntAC)
Source: Nat Commun. 2025 Jul 15;16:6498. doi: 10.1038/s41467-025-61692-3 (PMC12259957; doi:10.1038/s41467-025-61692-3)
Supplement: Supplementary file 2 — Description of Additional Supplementary Files [file 41467_2025_61692_MOESM2_ESM.pdf]

Description of Additional Supplementary Files

File Name: Supplementary Data 1
Description: Readcount data for *Drosophila* v.2 library, fitness screens with and without IntAC, and positive selection screens for cytidine and proaerolysin. This table contains log2 fold-change values and gene-level Z-scores calculated using MAGeCK for all sgRNAs and targeted genes in the genome-wide CRISPR knockout screens in Drosophila S2R+ cells. Screens include v.2 without anti-CRISPR and v.2 with anti-CRISPR (IntAC) conditions. Data include dropout screens for fitness gene identification and enrichment screens for cytidine (focused library) or proaerolysin (genome-wide library) resistance. Expression levels (FPKM) and gene annotations are also provided to facilitate comparison across screen conditions.

File Name: Supplementary Data 2
Description: *Drosophila* essential genes with 1-to-many human orthologs suggesting paralog masking. This table lists 123 Drosophila genes identified as essential in the v.2 IntAC screen that map to multiple human orthologs based on DIOPT scores. Each *Drosophila* gene is paired with its human ortholog(s) and their corresponding CERES scores from DepMap.
